# Refining the multimodality of semantic representations

**DOI:** 10.1016/j.tics.2025.10.014

**Published:** 2025-11-15

**Authors:** Laura Anna Ciaccio, Luca Rinaldi

**Affiliations:** 1 Department of Brain and Behavioral Sciences, University of Pavia , Pavia, Italy; 2 Cognitive Psychology Unit, IRCCS Mondino Foundation, Pavia, Italy

**Keywords:** LLMs, concepts, vision, sensorimotor, linguistic experience

## Abstract

A long-standing question in cognitive sciences concerns the specific contribution of linguistic and sensorimotor experience in shaping conceptual knowledge. A new study by Xu et al. shows that Large Language Models represent a powerful tool to advance this debate, helping to disentangle the relative contribution of different experiential modalities.

How concepts are formed and represented in the human mind remains a central question in cognitive sciences. Contemporary theories of semantic memory increasingly recognize the multimodal nature of semantic representations [1], highlighting the joint contributions of sensorimotor and linguistic experience. Accordingly, new conceptual knowledge can be acquired through direct sensory (e.g., seeing, tasting) and motor experiences (e.g., grasping, kicking), as well as from language alone, as, for instance, from patterns of word co-occurrence in linguistic input [see 2].

The advent of Large Language Models (LLMs) has apparently challenged this framework by showing that the amount of knowledge that can be inferred from language alone is by far greater than previously assumed. Indeed, recent research demonstrates that human ratings spanning several conceptual features (e.g., concreteness, valence, arousal) can be successfully reproduced by LLMs [3]. Notably, this holds even for domains that are traditionally considered highly sensory, such as color-adjective associations [4]. However, these findings offer only a partial view, leaving important questions unanswered. In fact, while these studies have focused solely on language, an approach that simultaneously addresses multiple modalities may be crucial to disentangle their respective contribution to shaping semantic knowledge. This is particularly important given that the relative weighting of sensorimotor and linguistic information is likely to vary with the nature of the concept, for instance, between more embodied concepts and more abstract ones (‘cheese’ vs. ‘democracy’).

A recent study by Xu and colleagues [5] provides crucial insights into these issues. The authors investigated to what extent semantic representations extracted from different state-of-the-art LLMs, namely GPT-3.5, GPT-4 (OpenAI), PaLM, and Gemini (Google), align with human representations. Critically, while GPT-3.5 and PaLM are trained on linguistic input only, GPT-4 and Gemini are pretrained on both textual and visual data (e.g., images). The LLMs were prompted to produce ratings for about 4,000 words on several conceptual dimensions. A distinction was made between sensorimotor and non-sensorimotor dimensions, depending on whether they directly assessed specific sensory or motor processes. Accordingly, the sensorimotor dimensions included association with the main sensory modalities (visual, auditory, haptic, olfactory, gustatory, interoceptive) and the main body effectors (foot/leg, hand/arm, mouth/throat, head excluding mouth, torso), while the non-sensorimotor dimensions included valence, dominance, arousal, size, gender, concreteness, and imageability. Model ratings were evaluated against existing databases of human ratings, both through analyses of individual dimensions and through a Representational Similarity Analysis (RSA) approach; for the RSA, the dimensions were grouped into broader subdomains (non-sensorimotor, sensory, motor) to examine their joint contribution to a concept’s representation. Both approaches revealed that the alignment between LLM-derived and human-derived ratings was strongest for the non-sensorimotor dimensions, weaker for sensory domains, and lowest for motor domains. Importantly, LLMs trained on both text and visual input (GPT-4 and Gemini) outperformed text-only models in capturing ratings pertaining to vision-related domains, particularly, imageability and concreteness [see also 6]. Taken together, these results provide evidence for a multimodal nature of semantic representations, indicating that relying on language alone yields impoverished representations for highly embodied domains, and specifically highlight the role of visual input in enriching semantic knowledge beyond linguistic experience.

A key avenue opened by the study of Xu et al. [5] concerns the extent to which other sensory modalities contribute to semantic representations over and above visual and linguistic input. Some studies, indeed, suggest that vision plays a dominant role among the senses, followed by audition, with taste and smell being comparatively neglected [7]. This raises the possibility that the cognitive system is selectively tuned to integrating only certain sensory experiences, while others do not meaningfully contribute to semantic knowledge. This gap can be addressed by leveraging large-scale computational models that selectively capture different sensory modalities, such as audition [8] or olfaction, to investigate whether some of them are better predictive of human data, thus revealing graded contributions of different modalities to conceptual knowledge (see Figure 1).

Closely related is the question of how deeply sensory experience is embedded within language itself, and to what extent the two can be disentangled [9,10]. If language already encodes rich sensory information, the boundary between linguistic and embodied representations becomes blurred. This, in turn, raises further questions: under what circumstances do models enriched with sensory input outperform text-only models? Put differently, when do speakers automatically retrieve direct sensory experience when accessing a semantic representation? Cultural and linguistic factors may also modulate the balance between linguistic and sensorimotor information in conceptual representation. For instance, in logographic languages such as Chinese, where characters correspond to whole units of meanings, are concepts more likely to embed sensory experience? Finally, what role do sound-symbolic forms play in bridging language and perception?

In sum, the study by Xu et al. shows that Large Language Models offer a powerful means to advance theories of semantic memory, while also raising new important questions for future research. Their findings and methodological innovations lay the groundwork for a comprehensive computational understanding of human conceptual knowledge.

## Figures and Tables

**Figure 1:**
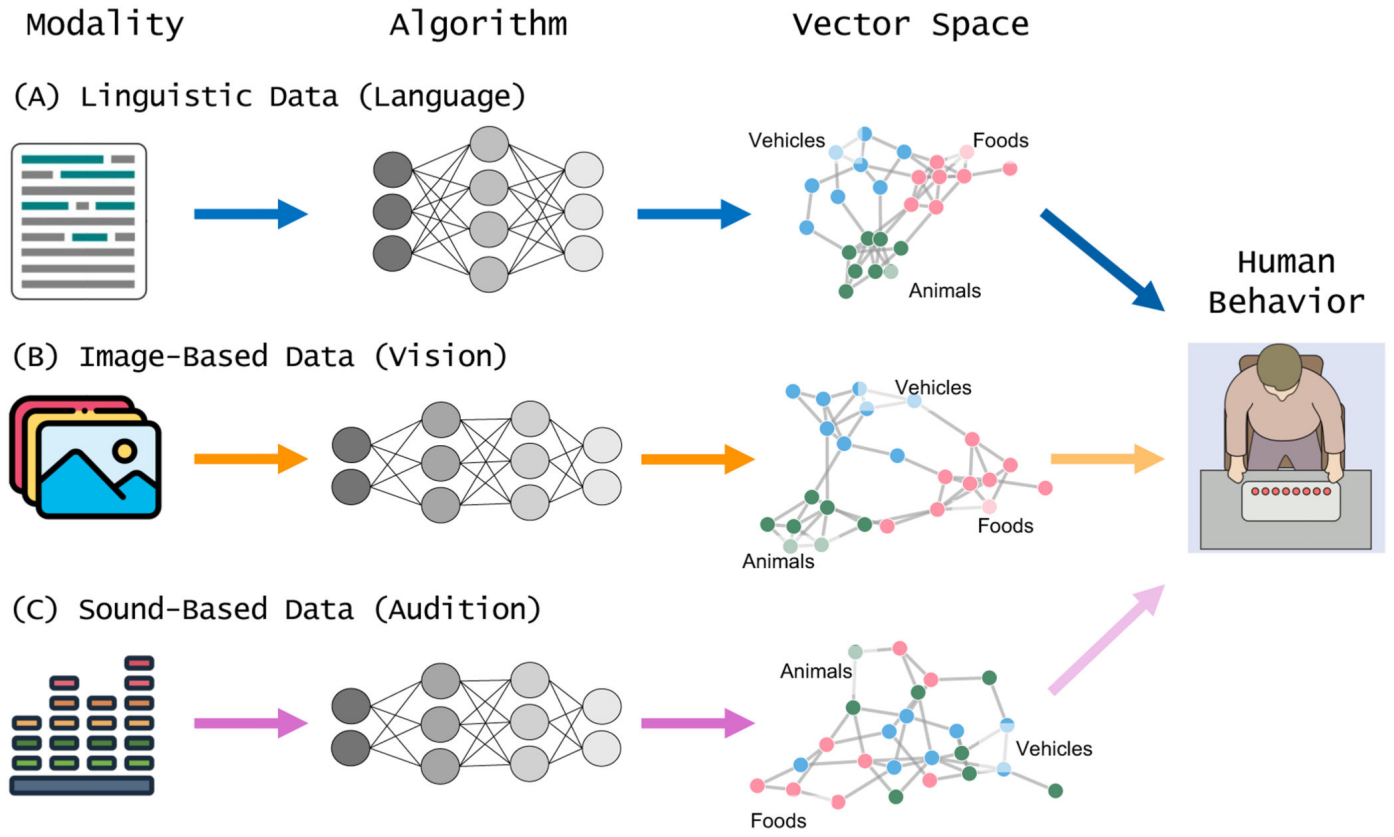
Implementation of a multimodal research workflow. Xu et al. [5] focused on the linguistic and visual modalities by leveraging large-scale computational models, such as LLMs trained on both text and visual input. Following a similar approach, one may rely on different computational models, such as distributional semantics models for language (A) and convolutional neural networks for vision (B), to generate concepts’ vector representations in the linguistic and visual domain [2]. (C) illustrates an extension of this framework to the auditory domain, in which the algorithm processes a large-scale database of sounds associated with specific concepts to produce vectors reflecting their prototypical auditory properties. Representations obtained at (A), (B), and (C) can then be used to predict human behavior. The expected outcome is the identification of different weighting across modalities (depicted by the arrows’ transparency gradient). These are likely to vary depending on the nature of the concept or task demands, reflecting the dynamic interplay among multiple experiential traces in conceptual representations.
